# The mitochondrial hinge protein, UQCRH, is a novel prognostic factor for hepatocellular carcinoma

**DOI:** 10.1002/cam4.1042

**Published:** 2017-03-23

**Authors:** Eun‐Ran Park, Sang‐Bum Kim, Jee‐San Lee, Yang‐Hyun Kim, Dong‐Hyoung Lee, Eung‐Ho Cho, Sun‐Hoo Park, Chul Ju Han, Bu‐Yeo Kim, Dong Wook Choi, Young Do Yoo, Ami Yu, Jae Won Lee, Ja June Jang, Young Nyun Park, Kyung‐Suk Suh, Kee‐Ho Lee

**Affiliations:** ^1^ Division of Radiation Cancer ResearchKorea Institute of Radiological and Medical SciencesSeoulKorea; ^2^ Department of Pathology and Brain Korea 21 PLUS Project for Medical ScienceYonsei University College of MedicineSeoulKorea; ^3^ Department of SurgeryKorea Institute of Radiological and Medical SciencesSeoulKorea; ^4^ Department of PathologyKorea Institute of Radiological and Medical SciencesSeoulKorea; ^5^ Department of Internal MedicineKorea Institute of Radiological and Medical SciencesSeoulKorea; ^6^ Herbal Medicine Research DivisionKorea Institute of Oriental MedicineDaejeonKorea; ^7^ Department of SurgerySamsung Medical CenterSungkyunkwan University School of MedicineSeoulKorea; ^8^ Laboratory of Molecular Cell BiologyGraduate School of MedicineKorea University College of MedicineSeoulKorea; ^9^ Korean Medicine Clinical Trial CenterKyung Hee University Korean Medicine HospitalSeoulKorea; ^10^ Department of StatisticsKorea UniversitySeoulKorea; ^11^ Department of PathologySeoul National University School of MedicineSeoulKorea; ^12^ Department of SurgerySeoul National University School of MedicineSeoulKorea; ^13^ Department of BiotechnologyCollege of Natural ScienceSeoul Women's UniversitySeoulKorea

**Keywords:** AFP, hepatocellular carcinoma, Mitochondria, Prognosis, UQCRH overexpression

## Abstract

Alterations in mitochondrial respiration contribute to the development and progression of cancer via abnormal biogenesis, including generation of reactive oxygen species. Ubiquinol–cytochrome c reductase hinge protein (UQCRH) consists of the cytochrome bc1 complex serving respiration in mitochondria. In the present study, we analyzed UQCRH abnormalities in hepatocellular carcinoma (HCC) and its association with clinical outcomes of patients. UQCRH expression in HCC was determined via semiquantitative and quantitative real‐time reverse transcriptase polymerase chain reaction of 96 surgically resected HCC tissues positive for hepatitis B virus surface antigen. UQCRH was frequently overexpressed in HCC tissues (46.8%, based on 2.1‐fold cutoff). UQCRH overexpression was observed in HCCs with larger tumor size, poorer differentiation, or vascular invasion. Kaplan–Meier analysis revealed significantly shorter overall (*P *=* *0.005) and recurrence‐free survival (*P *=* *0.027) in patients with tumors overexpressing UQCRH. The prognostic impact of UQCRH was significant in subgroups of patients divided according to the *α*‐fetoprotein (AFP) level. The patient subgroup with higher AFP levels (≥20 ng/mL) exhibited significant differences in 5‐year overall (18.5% vs. 67.9%) and recurrence‐free survival rates (11.1% vs. 46.4%) between groups with and without UQCRH overexpression. In contrast, no marked survival differences were observed between subgroups with lower AFP levels (<20 ng/mL). Multivariate analysis defined UQCRH as an independent poor prognostic factor. Conclusively, our results indicate that UQCRH overexpression is correlated with poor outcomes of HCC patients. Furthermore, in patients grouped as high risk based on elevated AFP, lack of UQCRH overexpression could be a useful indicator for clinical treatment.

## Introduction

Hepatocellular carcinoma (HCC) is the fifth most common cancer type and third most prevalent cause of cancer‐induced mortality worldwide 1. The majority of patients with HCC have a background of chronic liver disease induced by hepatitis B virus (HBV), hepatitis C virus (HCV), and alcoholic and nonalcoholic steatohepatitis 2, 3. Other risk factors for HCC include aflatoxin B1, diabetes, obesity, hemochromatosis, and metabolic diseases 2, 4, 5. Despite significant improvements in HCC management, including hepatectomy, liver transplantation, transarterial chemoembolization, and molecular‐targeted therapy, HCC patients continue to have high mortality and low survival rates 6, 7, 8. At present, prediction of HCC patient prognosis is generally based on clinical staging systems 9, 10. However, individuals with HCC at the same clinical stage display highly variable outcomes, indicating different biological behaviors of HCC subtypes, even at the same clinical stage 11, 12. Determination of effective prognostic markers for classifying HCC subtypes therefore remains an unmet medical need. Previously, we established microarray data sets for HCC dealt with patient prognosis 13, 14, 15. After validation, we present, for the first time, that ubiquinol–cytochrome c reductase hinge protein (UQCRH), a component of the cytochrome bc1 complex, classifies HCC patients according to clinical outcome.

UQCRH represents subunit 6 of the cytochrome bc1 complex present in the mitochondrial respiratory tract. This protein, also known as mitochondrial hinge protein, has a molecular weight of 11 kDa, and is essential for cytochrome c1 and cytochrome c complex formation 16, 17. The complex is composed of two *α* helices connected with two disulfide bridges and a highly acidic presequence 18, 19. UQCRH, a component of the respiration complex, is ubiquitously expressed in the majority of tissues, particularly organs associated with high‐energy metabolism 20. However, UQCRH association in human cancer has rarely been documented and its impact on patient prognosis never explored to date. While evidence of UQCRH abnormalities, either via structural rearrangement or promoter methylation, has been reported in human cancers, the potential association of this protein with human cancer remains to be elucidated.

For subtyping HCC, *α*‐fetoprotein (AFP), a well‐defined poor prognostic factor, has been combined and analyzed with alterations of other tumor‐associated genes 21, 22. Several prognostic biomarkers, including EpCAM, Villin 1, and CXCR7, are effective in subtyping AFP‐positive HCC 23, 24, 25. AFP influences angiogenesis and invasion through modulation of MMP2/9, VEGF, and VEGFR‐2 expression 26. Additionally, AFP regulates caspase‐3 and PI3K/AKT signaling pathways, in turn, influencing apoptotic death of HCC cells 27.

Abnormalities in mitochondrial proteins are proposed to affect HCC progression via dysregulation of bioenergetics and biosynthetics 28. Recently, we reported that Romo1, a ROS generator in mitochondria, promotes invasion, leading to poor prognosis, followed by the identification of another mitochondrial prognostic factor, UQCRH, as shown in the present study 29. High UQCRH expression in HCC was correlated with dismal patient survival. In addition, favorable outcomes for HCC patients were predicted in the group displaying no UQCRH overexpression, even in cases with abnormally elevated AFP.

## Materials and Methods

### Patients and tissue samples

HCC and adjacent liver tissue samples were obtained from patients diagnosed with HCC who received curative hepatic resection between 1992 and 2004 at Korea Cancer Center and Seoul National University Hospitals. The follow‐up period for survivors was over 5 years. We used frozen tissues of 96 tumor specimens, including 30 pair‐matched adjacent liver tissues, stored in liquid nitrogen. Ten normal liver tissues were collected from patients who underwent hepatic resection due to liver metastases from other primary tumors. This retrospective study was approved by the Institutional Review Boards of Korea Cancer Center Hospital and Seoul National University Hospital. Written informed consent was waived by the Institutional Review Board of Korea Cancer Center Hospital and obtained from patients of Seoul National University Hospital. Retrospectively collected clinicopathologic data were used to determine correlation of HCC with UQCRH expression.

### RNA extraction and cDNA synthesis

Total RNA was extracted from frozen tissue samples using the Qiagen RNeasy Mini Kit (Qiagen, Valencia, CA) according to the manufacturer's instructions. The concentration and quality of total RNA was measured with a NanoDrop ND‐1000 spectrophotometer (NanoDrop Technologies, Wilmington, DE) at 260 and 280 nm, and confirmed with an Agilent 2100 Bioanalyzer (Agilent Technologies, Santa Clara, CA). Total RNA was used as a template for complementary DNA synthesis. cDNA was synthesized using the 5× iScript reaction mixture including oligo (dT), random hexamer primers, and iScript reverse transcriptase (Bio‐Rad, Hercules, CA).

### Semiquantitative RT‐PCR and real‐time PCR

Reverse transcription polymerase chain reaction (RT‐PCR) was performed with the Maxime PCR PreMix Kit (iNtRON Biotechnology, Kyungi‐do, Korea). The following primer sequences were employed for semiquantitative RT‐PCR: UQCRH, 5′‐ATGGGACTGGAGGACGAGCA‐3′ (sense), 5′‐AAGAGTTTGTGGGCCACGCA‐3′ (antisense); RPS26, 5′‐TGCCTCCAAGATGACAAAGA‐3′ (sense), 5′‐TCCTTACATGGGCTTTGGTG‐3′ (antisense). Real‐time RT‐PCR was conducted using the iQ^™^ QSupermix (Bio‐Rad) and the CFX96 real‐time RT‐PCR detection system (Bio‐Rad). The primers and probe sequences used for real‐time RT‐PCR were: UQCRH, 5′‐GCAAAAGATGCTTACCGAATCCG‐3′ (sense), 5′‐CTCTCTCACTGTTGTTAGGGGATC‐3′ (antisense) and 5′‐FAM‐TCCTCCTCTTCCTCTTCCTCCTCCTCA‐BHQ‐1‐3′ (probe); 18S rRNA, 5′‐GGAGAGGGAGCCTGAGAAACG‐3′ (sense), 5′‐TTACAGGGCCTCGAAAGAGTCC‐3′ (antisense) and 5′‐FAM‐TACCACATCCAAGGAAGGCAGCAGGCG‐BHQ‐1‐3′ (probe). Reactions were assayed in triplicate. Relative UQCRH levels were normalized to median 18S rRNA expression. Relative UQCRH expression of HCC and adjacent liver tissues, compared to that of normal liver tissues, was analyzed using the comparative threshold cycle (2^−ΔΔC(t)^) method 30. Real‐time RT‐PCR for UQCRB, UQCRC2, cytochrome c1, and reference control RPS26 was conducted using the iQ^™^ SYBR^®^ Green Supermix (Bio‐Rad) and the CFX96 real‐time RT‐PCR detection system (Bio‐Rad). The primer sequences used for real‐time RT‐PCR were: UQCRB, 5′‐CCTTTATAATGACAGGATGTTTCGC‐3′ (sense), 5′‐TCTTTCCCGAATAACCTCTTTCAG‐3′ (antisense); UQCRC2, 5′‐GGCTCTAGTTGCTGGTTCTTAC‐3′ (sense), 5′‐GTCCCAAATTTCCACTTGCTG‐3′ (antisense); CYC1, 5′‐CAGCTACCATGTCCCAGATAG‐3′ (sense), 5′‐TGTGC CGCTTTATGGTGTAG‐3′ (antisense). RPS26, 5′‐CCAAGGACAAGGCCATTAAG‐3′ (sense), 5′‐AGCACCCGCAGGTCTAAATC‐3′ (antisense). Relative expression of each gene was normalized to median RPS26 expression.

### Western blotting analysis

Liver tissues were homogenized in lysis buffer (50 mmol/L Tris‐HCl [pH 7.4], 150 mmol/L NaCl, 5 mmol/L EDTA, 1% (v/v) Nonidet P‐40, 0.1% (w/v) sodium dodecyl sulfate, 0.5% [w/v] sodium deoxycholate) including protease inhibitor cocktail (P3100‐010, GenDEPOT, TX), incubated on ice for 20 min, and centrifuged for 20 min at 13,000 xg at 4°C. Approximately 20 *μ*g of total protein was subjected to 16% SDS‐PAGE and transferred to nitrocellulose blotting membrane. The membranes were blocked with 5% (w/v) skim milk in TBS‐T buffer (140 mmol/L NaCl, 25 mmol/L Tris‐HCl [pH 7.4], 2.7 mmol/L KCl, 0.05% [v/v] Tween 20) and incubated with the antibodies for UQCRH (ab134949, Abcam, Cambridge, UK) and *α*‐tubulin (sc‐5286, SantaCruz, CA) overnight at 4°C. After washing three times with TBS‐T, the membranes were incubated with a horseradish peroxidase‐conjugated secondary antibody (A120‐101P and A90‐116P, Bethyl Laboratories, TX) at room temperature for 1 h. The membrane was reacted with western blotting luminol reagent (sc‐2048, SantaCruz).

### Statistical analysis

Statistical analysis was performed using the SPSS software version 22 (SPSS Institute, Chicago, IL) and R package version 3.2.1 (http://cran.nexr.com/). All possible *P*‐values to discriminate between high‐ and low‐risk subgroups according to UQCRH expression were determined using the log‐rank test between two groups, and UQCRH expression ratios showing the lowest *P*‐value subsequently were selected as a cutoff point. Survival curves were plotted using a Kaplan–Meier method and differences in survival rates analyzed with a log‐rank test. Determination of the prognostic relevance of each variable for overall and recurrence‐free survival and multivariate analysis of prognostic factors were performed using the Cox regression model. Overall and recurrence‐free survival rates were calculated based on scoring of death or recurrence as the respective event. The starting point of follow‐up for survival analysis was the day of surgical resection. *P* < 0.05 were considered statistically significant.

## Results

### UQCRH is overexpressed in HCC

**Figure 1:**
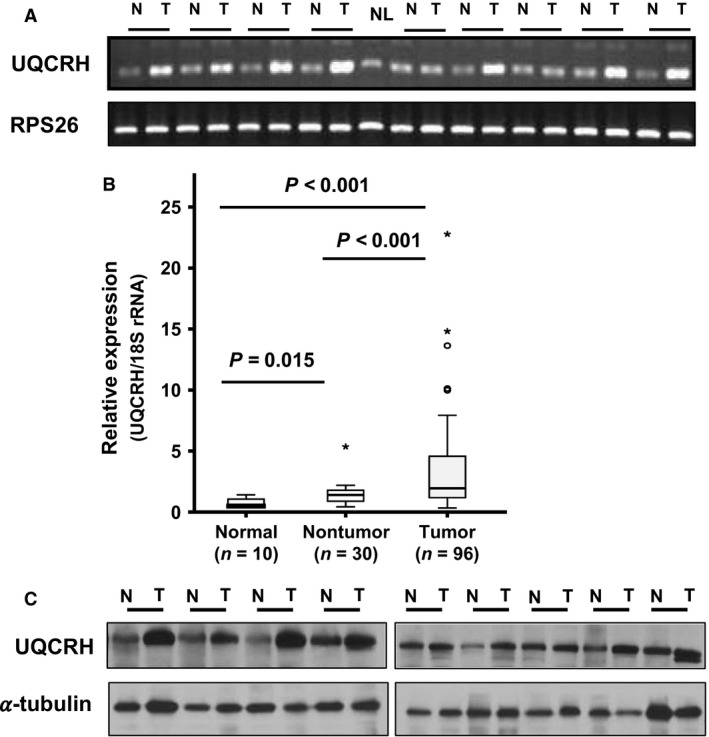
Overexpression of *UQCRH* in HCC. (A) *UQCRH* mRNA expression in HCC (T) and corresponding adjacent liver tissues (*N*), determined with semiquantitative RT‐PCR. NL indicates normal liver. (B) Box plot analysis illustrating differences in *UQCRH* mRNA expression levels between normal (*n* = 10), adjacent liver (*n* = 30), and HCC (*n* = 96) tissues. *UQCRH* expression was measured using real‐time RT‐PCR, with 18S rRNA as an internal control. (C) *UQCRH* protein level in pair‐marched HCC (T) and corresponding adjacent liver tissues (*N*) was determined by western blotting. *, More than 3/2 times of upper quartile.

To determine whether UQCRH expression is clinically associated with HCC, we initially measured mRNA levels in frozen HCC tissues that had been surgically removed. The experiment was performed using hepatitis B‐positive HCC specimens to minimize the effect of etiology. Semiquantitative RT‐PCR data revealed higher UQCRH expression in HCC than the corresponding adjacent liver tissues (Fig. 1A). As depicted in Figure S1A, three different UQCRH variants have been submitted to the NCBI database. Our real‐time RT‐PCR primers and TaqMan probe amplified only variant 1 representing wild‐type UQCRH in HCC tissues (detailed description in the legend to Fig. S1B). Under this condition, we quantitated UQCRH mRNA levels using an extended HBV‐positive HCC sample set (*n* = 96) (Table 1) and adjusted expression levels to that of normal liver tissue (*n* = 10). The adjacent liver tissues analyzed (*n* = 30) showed either background liver cirrhosis (*n* = 14) or fibrosis (*n* = 15). Real‐time RT‐PCR revealed significantly higher levels of UQCRH transcript in HCC tissues than adjacent liver tissues (*P *<* *0.001) (Fig. 1B), consistent with semiquantitative RT‐PCR findings. The adjusted mean levels of UQCRH expression in HCC and adjacent liver tissues were 3.27‐ and 1.47‐fold, respectively. Western blot analysis similarly disclosed higher UQCRH expression in HCC than adjacent liver tissues (Fig. 1C), clearly indicating that HCC tissues overexpress UQCRH.

**Table 1:** Patient demographics and pathologic data (*n* = 96)

Variables	Classification	Distribution
Gender	Male: Female	76:20
Age	Year, mean ± SD (range)	51.87 ± 9.48 (26–72)
Etiology	Hepatitis B:Hepatitis C	96: 00
AST^a^	IU/L, mean ± SD (range)	58.10 ± 47.03 (13–300)
ALT^b^	IU/L, mean ± SD (range)	51.48 ± 30.68(5–158)
AFP^c^	<20 ng/dL: 20 ng/dL≦	41: 54
Prothrombin (%)	<90: 90≦	46: 50
Child_P classification	A: B & C	81: 09
Total bilirubin	<1 mg/dL: 1 mg/dL≦	72: 24
Tumor size	cm, mean ± SD (range)	6.2 ± 3.6 (1.0–24.0)
Tumor number	Single: Multiple	77: 13
TNM stage	I:II: III: IV	24: 31: 22: 03
Tumor grade^d^	1: 2: 3: 4	13: 54: 29: 00
Fibrosis	No:Yes	06: 84
Cirrhosis	No:Yes	52: 38
Macroscopic vascular invasion	No:Yes	82: 11
Microscopic vascular invasion	No:Yes	63: 27
Capsule invasion	No:Yes	52: 43

a AST, aspartate aminotransferase.

b ALT, alanine transaminase.

c AFP, *α*‐fetoprotein.

d Edmonson–Steiner histological grade.

### UQCRH overexpression is associated with poor overall and recurrence‐free survival of HCC patients

**Figure 2:**
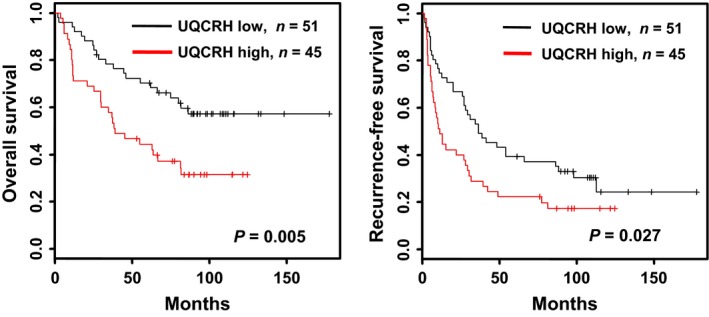
Kaplan–Meier survival analysis according to *UQCRH* expression and survival times in HCC patients. Kaplan–Meier curves showing overall and recurrence‐free survival were plotted according to relative *UQCRH* expression levels (low, black line <2.1‐fold and high, red line ≥2.1‐fold). The *P*‐value was determined using log‐rank analysis.

To determine whether UQCRH overexpression is associated with clinical outcome, we analyzed its mRNA levels in terms of patient survival. Patients were subdivided into two groups according to UQCRH mRNA levels to establish the suitable cutoff point of UQCRH overexpression as a prognostic factor. Subdivision was sequentially performed based on all possible patient combinations, and their prognostic *P*‐values plotted (Fig. S2). Statistically significant *P*‐values were sequentially aligned, among which 2.1‐fold (arrow) was the most significant in evaluating both overall and recurrence‐free survival. Using the 2.1‐fold cutoff point, Kaplan–Meier plot showed that patients with higher UQCRH expression (≥2.1‐fold) exhibited significant shorter overall (*P *=* *0.005) and recurrence‐free survival (*P *=* *0.027) than those with lower expression (<2.1‐fold) (Fig. 2). The 5‐year overall and recurrence‐free survival rates between the low‐ and high‐risk groups were 55.6% versus 29.4% and 77.8% versus 55.6%, respectively. Based on the 2.1‐fold cutoff point, UQCRH overexpression frequency was 46.8% in HCC. Our survival analyses indicate that UQCRH overexpression in mRNA level is associated with poor survival of HCC patients.

**Table 2:** Correlation between UQCRH expression and clinicopathological parameters (*n* = 96)

Variables	UQCRH Expression		*P* a
	<2.1‐fold	≦2.1‐fold	
Gender			
Male	38	38	0.232
Female	13	7	
Age (year)			
<52	19	24	0.114
≥52	32	21	
AFP (ng/mL)^b^			
<20	22	19	0.861
≥20	28	26	
AST (U/L)			
<40	27	15	0.053
≥40	24	30	
ALT (U/L)			
<35	16	16	0.664
≥35	35	29	
Child_P classification			
A	44	37	0.573
B,C	4	5	
Tumor size (cm)			
<3	11	3	0.035^d^
≥3	39	42	
Tumor grade^c^			
I	9	4	0.211
II, III	42	41	
Tumor number			
Single	37	40	0.015^d^
Multiple	11	2	
TNM stage			
I/II	29	26	0.544
III/IV	15	10	
Macroscopic vascular invasion			
No	47	35	0.061
Yes	3	8	
Microscopic vascular invasion			
No	34	29	0.854
Yes	14	13	
Capsule invasion			
No	30	22	0.389
Yes	21	22	
Cirrhosis			
No	29	23	0.588
Yes	19	19	

a Significance of *UQCRH* overexpression in association with clinicopathological parameters was calculated using chi‐square test.

b AFP, *α*‐fetoprotein; AST, aspartate aminotransferase; ALT, alanine transaminase.

c Edmonson–Steiner histological grade.

d *P* <0.05 is considered statistically significant.

### UQCRH overexpression is a particularly effective indicator in patients subgrouped according to AFP levels

Next, we focused on defining the clinical parameters that affect the prognostic significance of UQCRH. To this end, the correlation of UQCRH with survival was further analyzed after subdividing patients according to clinicopathological parameters, including AFP, tumor grade, tumor size, vascular invasion, and cirrhosis. Among the patient subgroups, those classified based on AFP level (<20 and ≥20 ng/mL) and tumor grade (I and II, III) were significantly associated with UQCRH overexpression (Table S1). In contrast, tumor size (<3 and ≥3 cm), vascular invasion (yes and no), and cirrhosis (yes and no) were not very effective in classifying the patients based on UQCRH mRNA levels. Due to the limited number of patients (*n* = 9) belonging to grade I, we thus focused on subgroups classified by AFP. When the cutoff value for AFP was gradually increased, the prognostic significance of UQCRH was consistently observed only in patient subgroups with higher AFP levels (≥ specific cutoff) (Table S2).

**Figure 3:**
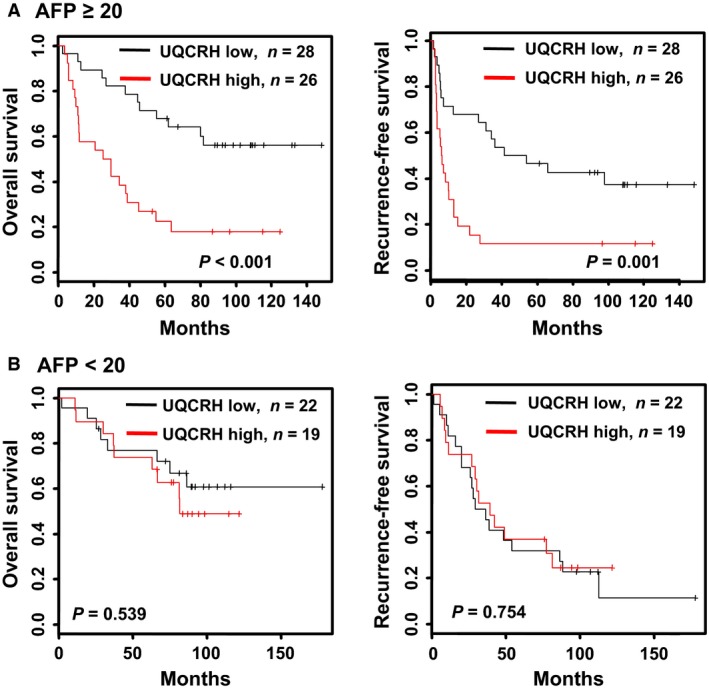
*UQCRH* expression‐based Kaplan–Meier survival analysis in HCC patients stratified by AFP level. Kaplan–Meier survival curves of HCC patient subgroups subdivided by AFP levels ≥20 ng/mL (A) and <20 ng/mL (B), based on *UQCRH* expression. Overall and recurrence‐free survival in the two subgroups were analyzed according to relative *UQCRH* expression (low, black line <2.1‐fold and high, red line ≥2.1‐fold). *P*‐values were determined using log‐rank analysis.

**Figure 4:**
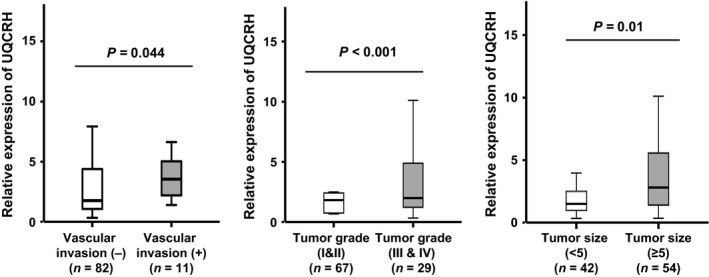
HCC tissues associated with upregulation of UQCRH. HCC patient tissues (*n* = 96) were subdivided in relation to clinicopathological parameters as described in Table 2, and the mean values of UQCRH expression compared between the subgroups. HCC subgroups showing significant differences in UQCRH expression are presented.

In patients with higher AFP (≥20 ng/mL), the prognostic value of UQCRH mRNA was markedly elevated, but was not significant in those with lower AFP levels (<20 ng/mL). The 5‐year overall and recurrence‐free survival rates of patients (AFP≥20 ng/mL) without and with UQCRH overexpression were 67.9% versus 18.5% and 46.4% versus 11.1%, respectively (Fig. 3). AFP is a well‐known poor prognostic factor for HCC. Thus, HCC patients without UQCRH overexpression are expected to show favorable outcomes, despite high levels of AFP (≥20 ng/mL). Further analysis of the correlation between clinicopathological parameters and UQCRH overexpression (based on the 2.1‐fold cutoff) revealed that UQCRH overexpression is associated with tumor size (*P *=* *0.035) and tumor number (*P *=* *0.015) (Table 2). In addition, HCC tissues with larger tumor size (≥5 cm), poorer grade (III and IV), or macroscopic vascular invasion were associated with upregulation of UQCRH expression (Fig. 4), indicating that UQCRH is abundant in aggressive HCC.

### UQCRH is an independent poor prognostic factor for HCC

To further assess the association of UQCRH with clinical outcomes, we performed univariate and multivariate Cox regression analyses in relation to patient survival. Based on univariate Cox regression analysis without subdivision (*n* = 96), UQCRH overexpression had a significant adverse influence on overall (*P *=* *0.006) and recurrence‐free survival (*P *=* *0.029) in our patient cohort, together with several clinicopathological parameters, including tumor stage (*P *=* *0.007 and *P *=* *0.027), macroscopic vascular invasion (*P *<* *0.001 and *P *=* *0.001), and capsule invasion (*P *=* *0.002 and *P *=* *0.033) (Table S3). UQCRH significance was also observed in multivariate analysis with a forward entry of parameters (*P *<* *0.05) (Table 3). With the multivariate approach, macroscopic vascular invasion was the clinical parameter affecting both overall (HR, hazard ratio=3.170, *P *=* *0.002) and recurrence‐free survival (HR* *=* *3.067, *P *=* *0.001), while capsule invasion (HR=2.253, *P *=* *0.008) affected overall survival only. In this setting, UQCRH overexpression was an independent poor prognostic factor (HR* *=* *2.271, *P *=* *0.007 for overall survival and HR* *=* *1.621, *P *=* *0.049 for recurrence‐free survival). In patient subgroups with AFP levels ≥20 ng/mL, UQCRH had a profound effect in both univariate (Table S4) and multivariate analyses (Table 4). The consequent multivariate HRs of UQCRH overexpression on overall and recurrence‐free survival were 2.519 (*P *=* *0.027) and 2.252 (*P *=* *0.026), respectively. Therefore, UQCRH overexpression serves as a high‐risk factor for HCC cases, especially those with high levels of AFP.

**Table 3:** Multivariate analysis of UQCRH expression and clinicopathological parameters (*n* = 89)

Variables	HR^b^	95% CI^c^	*P* a
Overall survival (*n* = 89)			
Capsule invasion	2.253	1.233–4.116	0.008
Macroscopic vascular invasion	3.170	1.514–6.637	0.002
UQCRH expression	2.271	1.252–4.120	0.007
Recurrence‐free survival (*n* = 89)			
Macroscopic vascular invasion	3.067	1.573–5.981	0.001
UQCRH expression	1.621	1.003–2.625	0.049

a Significance determined by multivariate Cox regression analysis.

b HR, hazard ratio.

c CI, confidence interval.

**Table 4:** Multivariate analysis of UQCRH expression and clinicopathological parameters in patient subgroup (AFP ≥ 20, *n* = 51)

Variables	HR^a^	95% CI^b^	
Overall survival			
Macroscopic vascular invasion	4.712	1.840–12.065	0.001
Capsule invasion	2.511	1.165–5.408	0.019
UQCRH overexpression	2.519	1.112–5.706	0.027
Recurrence‐free survival			
Macroscopic vascular invasion	2.729	1.123–6.629	0.027
UQCRH overexpression	2.252	1.102–4.603	0.026

Significance determined by multivariate Cox regression analysis.

a HR, hazard ratio.

b CI, confidence interval.

### UQCRH is upregulated concurrently with the mitochondrial components UQCRB, UQCRC2, and cytochrome c1

**Figure 5:**
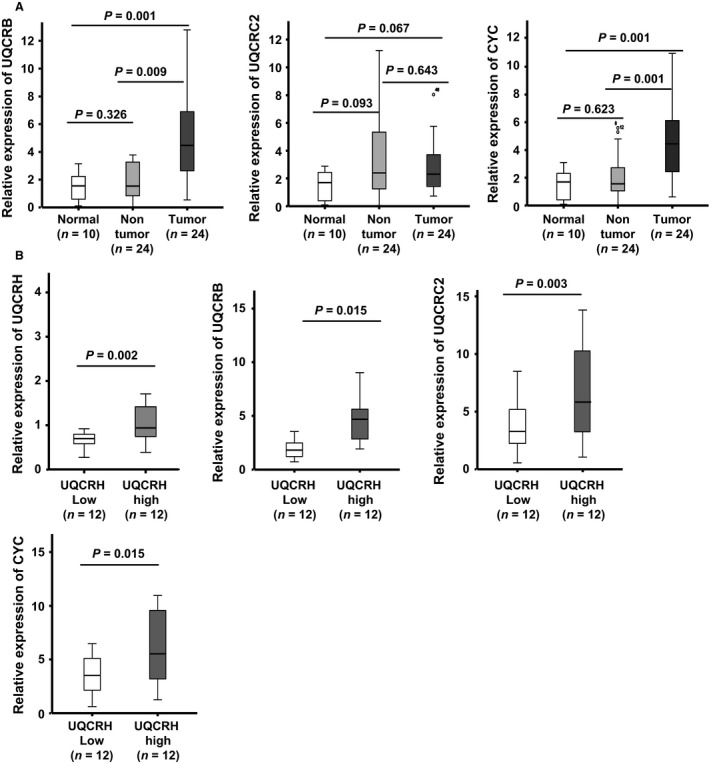
Correlation between UQCRH overexpression and expression of complex III components. (A) Expression levels of other complex III components (UQCRB, UQCRC2, and cytochrome c1) were examined in normal (*n* = 10), adjacent liver (*n* = 24), and HCC (*n* = 24) tissues. (B) Tissue samples were subdivided into two groups according to UQCRH expression: high (*n* = 12) and low (*n* = 12), and expression of complex III components examined in the subdivided groups using real‐time RT‐PCR.

Mitochondrial dysfunction is crucial in the progression of cancers, including HCC. Interestingly, a decrease in mitochondrial number in HCC is ultimately associated with poor patient outcomes 31, 32. It is speculated that UQCRH overexpression occurs together with other mitochondrial components as a compensatory mechanism against reduction of mitochondria. To explore this possibility, we determined the expression levels of three complex III components, UQCRB, UQCRC2, and cytochrome c1 in normal, nontumor adjacent liver tissues, and HCC tissues. Notably, UQCRB (*P *=* *0.001), UQCRC2 (*P *=* *0.067), and cytochrome c1 (*P *=* *0.001) were overexpressed in HCC, compared to normal liver tissues (Fig. 5A). We further subdivided into two groups according to UQCRH expression: high (*n* = 12) and low (*n* = 12). Similar to UQCRH (*P *=* *0.002), expression levels of the components were also different between the groups (UQCRB, *P *=* *0.015; UQCRC2, *P *=* *0.003; cytochrome c1, *P *=* *0.015, Fig. 5B). Our findings indicate that UQCRH is overexpressed in conjunction with UQCRB, UQCRC2, and cytochrome c1 in HCC.

## Discussion

HCC cases with large tumors, macroscopic vascular invasion, and/or increased AFP levels in serum are associated with early recurrence, and consequently, dismal clinical outcomes. However, high‐risk patients can survive longer than expected, despite these clinical conditions. In the present study, UQCRH was identified as a novel poor prognostic factor for HCC, especially in patients with high serum AFP levels.

UQCRH is associated with mitochondrial respiration and energy metabolism 33. Matrix‐assisted laser desorption/ionization time‐of‐flight (MALDI‐TOF) data indicate that UQCRH plays a role in calorie restriction and age 34. However, UQCRH has rarely been investigated in relation to cancer. Modena et al. reported that the UQCRH gene structurally comprises two distinct genes, EWS and ZSG, and subsequently inactivated with the occurrence of a premature stop codon between the exons. The group additionally demonstrated downregulation of UQCRH expression in limited number of breast and ovarian cancer cell lines through a mechanism of promoter inactivation via epigenetic methylation 20. The interpretations and conclusions of these authors are not in line with our observation that UQCRH is overexpressed in HCC. These earlier findings were interpreted based on comparison of UQCRH transcripts between the cancer cell lines, in contrast to our approach whereby UQCRH expression was compared between pair‐matched HCC and corresponding adjacent liver tissues. Indeed, we also observed low levels of UQCRH transcripts among some of the HCC cell lines examined, similar to normal cells (data not shown). Therefore, the earlier observations need to be interpreted with caution. Similar to previous findings, Owens and coworkers demonstrated UQCRH overexpression in various human cancer tissues through analysis of the Oncomine database, including myeloma, lung, prostate, brain, and bladder cancers, and further validated overexpression in breast cancer tissues 35. Recently, Gao et al. reported UQCRH protein overexpression in tumor tissues and sera in lung adenocarcinoma patients 36. While these reports are consistent with our findings, controversial data were obtained from an immunohistochemical study on clear cell renal carcinoma, showing that UQCRH protein expression is relatively lower in tumor than nontumor regions, with association between low levels in tumors and poor prognosis of patients 37. These previous findings as well as our present results are based on extensive and careful analyses. The differences in UQCRH levels in cancer tissues between studies may reflect variable expression according to cancer type. Our real‐time RT‐PCR, semiquantitative RT‐PCR and western blot analyses clearly and consistently revealed UQCRH overexpression in HCC. In addition, the UQCRH antibody used clearly detected a single band representing UQCRH in several cell lines on a blot (Fig. S3), further supporting our western blot results (Fig. 1C) showing higher UQCRH protein levels in cancer than nontumor adjacent liver tissues. Collectively, the prior and present findings suggest that UQCRH expression is normally suppressed by promoter methylation, whereas in HCC, demethylation occurs and ultimately leads to overexpression of UQCRH. Initially, hinge protein overexpression was reported in a prostate cancer cell line 38.

Abnormalities in the components and number of mitochondria influence the progression and prognosis of HCC. Increased reduction in mitochondrial number is associated with poorer outcomes of HCC 31, 32. As demonstrated in the current study, UQCRH mRNA was upregulated, together with those of other complex III components, UQCRB, UQCRC2, and cytochrome c1, in HCC. While we were unable to examine the levels of all mitochondrial components and establish a correlation between their expression patterns and mitochondrial numbers in HCC, our results support the possibility that high expression of UQCRH, in conjunction with other mitochondrial components, presents a compensatory mechanism against the decrease in mitochondria number.

In conclusion, extensive analysis of intratumoral UQCRH mRNA expression patterns in relation to various clinicopathological parameters revealed that overexpression of UQCRH mRNA in tumor tissues is significantly associated with poor overall and recurrence‐free survival in HCC patients. Interestingly, the prognostic significance of UQCRH was most pronounced in patients with higher levels of AFP (>20 ng/mL). Large amounts of AFP in patient serum are correlated with aggressive characteristics of HCC 39, 40. In fact, patients with high AFP levels experience frequent vascular invasion and intrahepatic metastasis 41, 42. Accordingly, AFP levels represent a valuable index to evaluate overall and recurrence‐free survival 43, 44. In our subgroup analysis according to AFP status, UQCRH expression was advantageous for further classifying patients considered the high‐risk group due to elevated serum AFP levels. Considering our data together with the prior finding that high serum levels of UQCRH protein are associated with poor prognosis in lung cancer patients 36, we propose that detectable UQCRH in serum provides a useful diagnostic and prognostic marker to classify HCC patients according to risk. In particular, in patients estimated as high risk due to serum AFP, lack of UQCRH overexpression may be an indication for clinical treatment, serving as a clinically useful prognostic factor.

## Conflict of Interest

The authors declare no conflicts of interest.

## Supporting information


**Figure S1.** Analysis of UQCRH variants. (**A**) Schematic diagram showing exon and intron organization in three different variants of UQCRH enrolled in the NCBI database.
**Figure S2.** *P*‐value plots showing all possible combinations of two patient groups according to *UQCRH* expression.
**Figure S3.** Exogenous expression of UQCRH in several cell lines. UQCRH or empty vector was transfected into HeLa, 293T, and Huh7 cells.

**Table S1.** Identification of clinical parameters effective for survival discrimination based on UQCRH expression.
**Table S2.** Effect of AFP levels in survival discrimination based on UQCRH expression.
**Table S3.** Univariate analysis of UQCRH expression and clinicopathological parameters (*n* = 96).
**Table S4.** Univariate analysis of UQCRH expression and clinicopathological parameters in patient subgroup (AFP ≥ 20, *n* = 54).
