# Supplementary material for: The mitochondrial hinge protein, UQCRH, is a novel prognostic factor for hepatocellular carcinoma
Source: Cancer Med. 2017 Mar 23;6(4):749–60. doi: 10.1002/cam4.1042 (PMC5387164; doi:10.1002/cam4.1042)
Supplement: Supplementary file 1 — Figure S1. Analysis of UQCRH variants. (A) Schematic diagram showing exon and intron organization in three different variants of UQCRH enrolled in the NCBI database. Figure S2. P‐value plots showing all possible combinations of two patient groups according to UQCRH expression. Figure S3. Exogenous expression of UQCRH in several cell lines. UQCRH or empty vector was transfected into HeLa, 293T, and Huh7 cells. [file CAM4-6-749-s001.pdf]

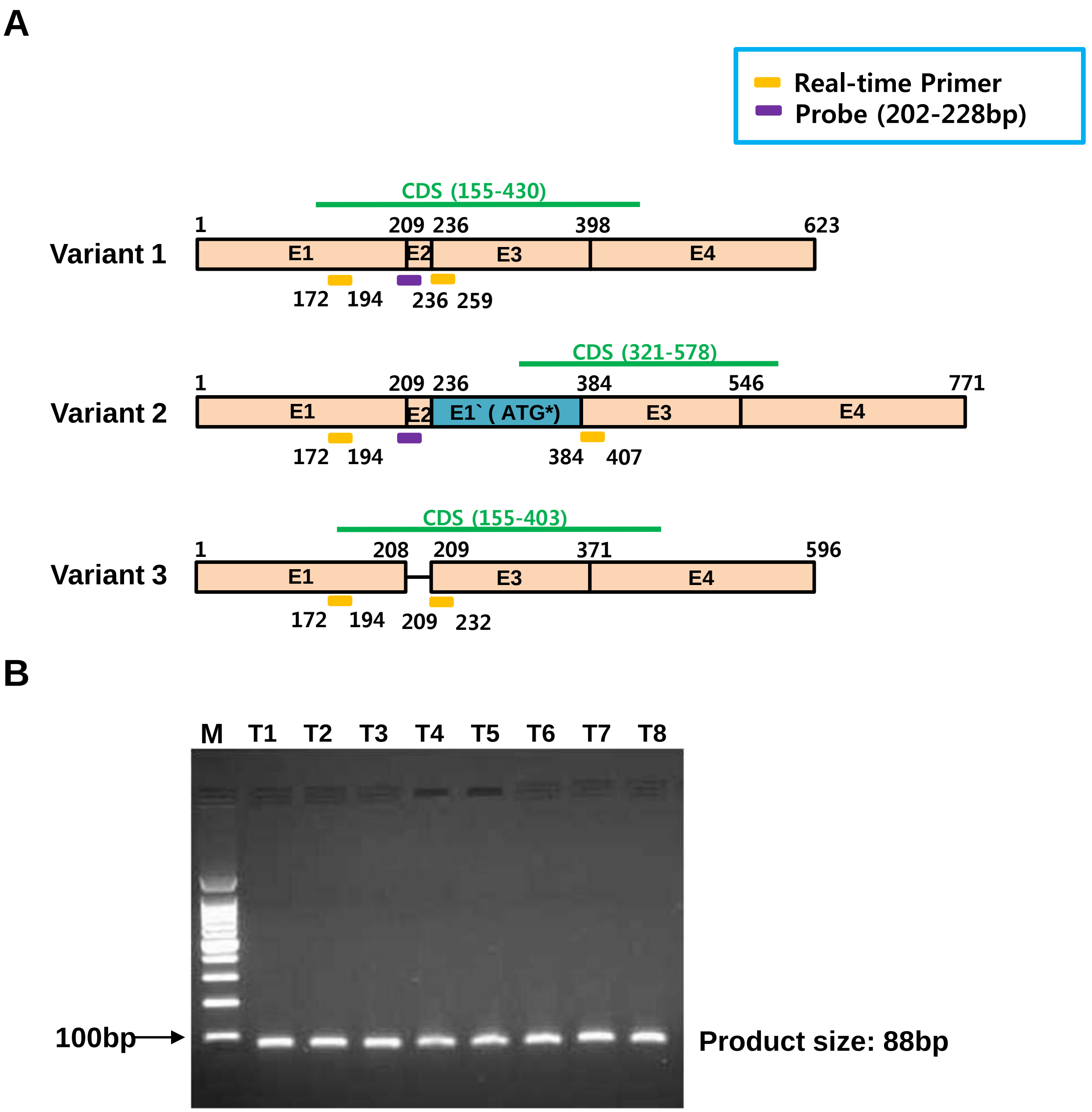

Supplementary Figure 1. Analysis of UQCRH variants. (**A**) Schematic diagram showing exon and intron organization in three different variants of UQCRH enrolled in the NCBI database. Variant 1 is wild-type UQCRH composed of exons 1 to 4, variant 2 contains an alternate internal exon between exons 2 and 3, and variant 3 is devoid of exon 2. The numbers on the diagrams for each UQCRH variant indicate the location on the mRNA sequence. The filled bars signify upstream and downstream primers marked in numbers on each mRNA sequence. The blank bar denotes the Taqman probe. (**B**) Final real-time PCR products of eight different HCC tissues were run on an agarose gel and visualized with ethidium bromide. Our real-time RT-PCR primers and TaqMan probe designed detected only variants 1 and 2 with product sizes of 88 and 236 bp, respectively. Variant 3 lacking exon 2 could not be amplified, since the TaqMan probe specifically recognizes exon 2. Upon visualization of real-time RT-PCR products in eight different tissues, only the 88 bp band was detected, indicating amplification of wild-type variant 1.

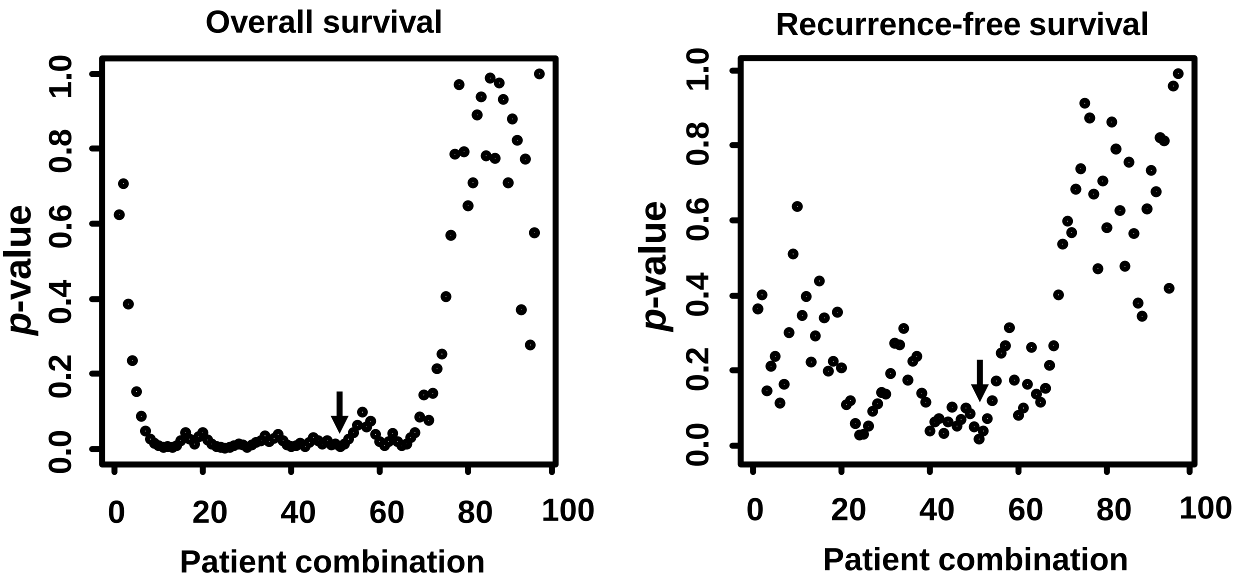

Supplementary Figure 2. *p*-value plots showing all possible combinations of two patient groups according to *UQCRH* expression. HCC patients were aligned according to *UQCRH* expression levels in ascending order, and log rank *p*-values determined by comparing the two groups. The arrow indicates the cut-off point (2.1-fold) showing the lowest *p*-value for overall and recurrence-free survival (51th patient).

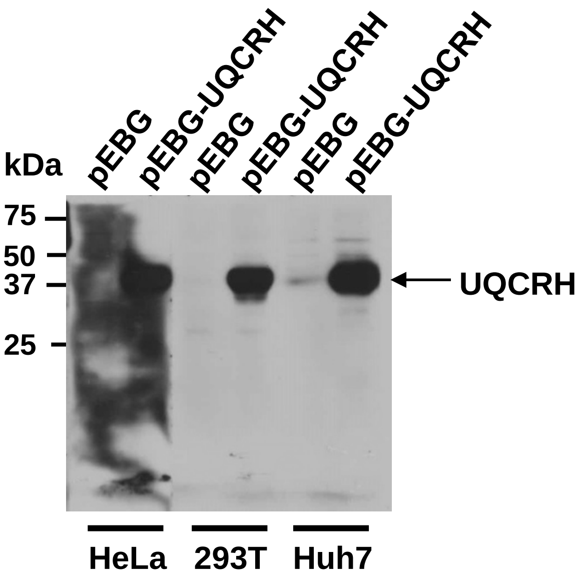

Supplementary Figure 3. Exogenous expression of UQCRH in several cell lines. UQCRH or empty vector was transfected into HeLa, 293T and Huh7 cells. After 48 h, protein samples were separated on a 16% SDS PAGE-gel. The UQCRH protein was detected as a single band.
