# Supplementary material for: The mitochondrial hinge protein, UQCRH, is a novel prognostic factor for hepatocellular carcinoma
Source: Cancer Med. 2017 Mar 23;6(4):749–60. doi: 10.1002/cam4.1042 (PMC5387164; doi:10.1002/cam4.1042)
Supplement: Supplementary file 2 — Table S1. Identification of clinical parameters effective for survival discrimination based on UQCRH expression. Table S2. Effect of AFP levels in survival discrimination based on UQCRH expression. Table S3. Univariate analysis of UQCRH expression and clinicopathological parameters (n = 96). Table S4. Univariate analysis of UQCRH expression and clinicopathological parameters in patient subgroup (AFP ≥ 20, n = 54). [file CAM4-6-749-s002.pdf]

Supplementary Table 1. Identification of clinical parameters effective for survival discrimination based on UQCRH expression

Variables	Classification	*P-value**	
		**Overall survival**	**Recurrence-free survival**
AFP	<20	0.539	0.754
	≥20	**<0.001****	**0.001**
Tumor grade	I,	0.840	0.388
	II, III	**0.008**	**0.010**
Tumor size (cm)	<3	0.961	0.468
	≥3	**0.010**	0.136
Vascular invasion	no	**0.031**	0.116
	yes	0.106	0.200
Cirrhosis	no	0.155	0.436
	yes	0.052	0.050

*Significance determined by log-rank test.

** Bold indicates *p*<0.05.

Supplementary Table 2. Effect of AFP levels in survival discrimination based on UQCRH expression

AFP	Distribution	*P-value**	
		Overall survival	Recurrence-free survival
**<20**	42	0.539	0.754
**≥20**	54	**<0.001****	**0.001**
**<50**	50	0.562	0.641
**≥50**	46	**<0.001**	**<0.001**
**<100**	60	0.296	0.994
**≥100**	36	**<0.001**	**<0.001**
**<200**	64	0.122	0.745
**≥200**	32	**0.001**	**<0.001**
**<400**	68	**0.041**	0.413
**≥400**	28	**0.007**	**0.002**

*Significance determined by log-rank test.

** Bold indicates $p<0.05$.

Supplementary Table 3. Univariate analysis of UQCRH expression and clinicopathological parameters (n=96)

Factor***	Overall survival			Recurrence-free survival		
	HR**	95% CI***	*P-value**	HR**	95% CI**	*P-value**
Gender (F vs M)	0.641	0.301 ~ 1.365	0.249	0.569	0.299 ~ 1.082	0.086
Age (≥52 vs <52)	1.084	0.622 ~ 1.886	0.777	1.277	0.801 ~ 2.036	0.304
AFP (≥20 vs <20)	1.890	1.051 ~ 3.399	**0.033******	1.273	0.798 ~ 2.030	0.311
AST (≥40 vs <40)	1.673	0.941 ~ 2.974	0.080	1.186	0.747 ~ 1.884	0.469
ALT (≥35 vs <35)	0.961	0.537 ~ 1.720	0.893	0.825	0.512 ~ 1.329	0.429
Child_P classification (B or C vs A)	2.211	0.989 ~ 4.945	0.053	1.607	0.732 ~ 3.528	0.237
Tumor size (≥3 vs <3)	1.854	0.736 ~ 4.670	0.190	2.360	1.081 ~ 5.156	**0.031**
Tumor number (multiple vs solitary)	1.072	0.480 ~ 2.391	0.866	1.262	0.661 ~ 2.413	0.481
Tumor grade (II or III vs I)	2.962	0.922 ~ 9.517	0.068	0.798	0.419 ~ 1.522	0.494
TNM stage (III or IV vs I or II)	2.288	1.256 ~ 4.167	**0.007**	1.811	1.069 ~ 3.065	**0.027**
Macroscopic vascular invasion (yes vs no)	3.859	1.887 ~ 7.888	**<0.001**	2.967	1.535 ~ 5.734	**0.001**
Microscopic vascular invasion (yes vs no)	1.989	1.112 ~ 3.556	**0.020**	1.380	0.826 ~ 2.306	0.218
Capsule invasion (yes vs no)	2.504	1.416 ~ 4.427	**0.002**	1.656	1.041 ~ 2.634	**0.033**
Cirrhosis (yes vs no)	1.570	0.896 ~ 2.752	0.115	1.363	0.842 ~ 2.208	0.208
UQCRH expression (≥2.1 vs <2.1)	2.189	1.25 ~ 3.834	**0.006**	1.673	1.054 ~ 2.655	**0.029**

*Significance determined by Univariate Cox regression analysis

HR, hazard ratio; ***CI, confidence interval; ** Bold indicates $p < 0.05$.

Supplementary Table 4. Univariate analysis of UQCRH expression and clinicopathological parameters in patient subgroup (AFP≥20, n=54)

Variables	Overall survival			Recurrence-free survival		
	**HR****	**95% CI*****	***P-value****	**HR****	**95% CI*****	***P-value****
Gender (F vs M)	0.475	0.196 ~ 1.1547	0.100	1.491	0.792 ~ 2.807	0.216
Age (≥52 vs <52)	1.307	0.655 ~ 2.609	0.448	0.550	0.253 ~ 1.195	0.131
AST (≥40 vs <40)	1.201	0.558 ~ 2.585	0.640	1.265	0.630 ~ 2.541	0.508
ALT (≥35 vs <35)	0.667	0.323 ~ 1.378	0.274	0.693	0.357 ~ 1.345	0.278
Child_P classification (B or C vs A)	2.236	0.908 ~ 5.505	0.080	1.690	0.700 ~ 4.084	0.244
Tumor size (≥3 vs <3)	2.066	0.629 ~ 6.781	0.232	3.326	1.021 ~ 10.837	**0.046*******
Tumor number (multiple vs solitary)	0.427	0.102 ~ 1.794	0.245	0.865	0.306 ~ 2.447	0.785
Tumor grade (II or III vs I)	3.364	0.459 ~ 24.636	0.233	0.462	0.163 ~ 1.304	**0.145**
TNM stage (III or IV vs I or II)	1.405	0.651 ~ 3.032	0.387	1.554	0.768 ~ 3.144	0.220
Macroscopic vascular invasion (yes vs no)	5.135	2.141 ~ 12.315	**<0.001**	3.614	1.554 ~ 8.404	**0.003**
Microscopic vascular invasion (yes vs no)	1.753	0.832 ~ 3.693	0.140	1.443	0.723 ~ 2.878	0.298
Capsule invasion (yes vs no)	2.471	1.199 ~ 5.091	**0.014**	2.048	1.076 ~ 3.898	**0.029**
Cirrhosis (yes vs no)	0.854	0.402 ~ 1.815	0.682	0.947	0.484 ~ 1.852	0.873
UQCRH expression (≥2.1 vs <2.1)	3.419	1.659 ~ 7.046	**0.001**	2.801	1.462 ~ 5.367	**0.002**

*Significance determined by Univariate Cox regression analysis

HR, hazard ratio; ***CI, confidence interval; ** Bold indicates $p < 0.05$.
